# Comparison of locking plates and intramedullary nails in treatment of three-part or four-part proximal humeral neck fractures in elderly population: A randomized trial protocol

**DOI:** 10.1097/MD.0000000000022914

**Published:** 2020-11-13

**Authors:** Hua Song, Mingming Wang, Hongyang Du, Weidong Mu

**Affiliations:** a School of Medicine, Shandong University; b Department of Orthopaedics, Tengzhou Central People's Hospital; c Department of Orthopaedics, Shandong Provincial Hospital Affiliated to Shandong University, Shandong, China.

**Keywords:** Three or four-part, intramedullary nail, locking plate, proximal humeral neck fractures, study protocol

## Abstract

**Background:**

Locking plate and intramedullary nail are two commonly applied methods to fix proximal humeral fractures. There are limited randomized studies that specifically evaluate the results of proximal humeral neck fractures with three-part or four-part treated by locking plates or intramedullary nails. Our goal was to compare functional outcomes, complications, and imaging features between the two groups.

**Methods:**

This single-center, prospective, randomized controlled test will be conducted in Tengzhou Central People's Hospital. Patients with these conditions will be included: age between 55 and 80 years; are able to communicate normally and agree to participate in our study; with the radiological evidence of proximal humeral fractures with three-part or four-part; surgical treatment was performed within twenty-one days after the acute fracture. Consecutive patients with proximal humeral fractures will be stochastic to be dealt with a locking plate or a bone nail. The informed consent will be acquired in each patients. Two groups will use the same postoperative rehabilitation protocol. Clinical outcomes include Intraoperative blood loss, operation time, Constant-Murley score, Disability, Arm, Shoulder and Hand score, shoulder range of motion (such as external rotation), and postoperative complications. The significance level was defaulted as *P* < .05.

**Results:**

This study will provide a solid theoretical basis for exploring which technique is better in treatment of 3-part or 4-part proximal humeral neck fractures in elderly population.

**Trial registration:**

This study protocol was registered in Research Registry (number: researchregistry6047).

## 1 Introduction

Fractures of proximal humerus mostly occur in the elderly. With the aging of population becoming more and more serious, the incidence rate has raised by nearly 3 times in recent 30 years.^[1–3]^ Especially the three-part and four-part of proximal humerus fractures in the elderly are extremely difficult to treat. There are a variety of treatments for displaced and unstable proximal humeral fractures. It is difficult for the medical community to reach a consensus on the optimal treatment of injuries.^[4–8]^

Locking plate and intramedullary nail are two commonly applied methods to fix proximal humeral fractures. With the development of science, the biological stability of steel plate and nail is equally excellent.^[9–14]^ However, although the stability of plate fixation is high, the range of operation is large, which increases the risk of ischemic necrosis. The patient's rotator cuff may be torn, and the function of the nail may be damaged. Complications such as implant impact and secondary screw perforation are well known.^[15–18]^

In current literature, there are limited randomized studies that specifically evaluate the results of proximal humeral neck fractures with three-part or four-part treated by locking plates or intramedullary nails. We thus designed a prospective, randomized controlled trial of 2 groups of patients treated with intramedullary nails and locking plates for three-part or four-part proximal humeral neck fractures in patients between 55 and 80 years. Our goal was to compare functional outcomes, complications, and imaging features between the two groups.

## 2 Materials and methods

### 2.1 Participants

Patients with these conditions will be included: age between 55 and 80 years; are able to communicate normally and agree to participate in our study; with the radiological evidence of proximal humeral fractures with three-part or four-part; surgical treatment was performed within 21 days after the acute fracture. The excluding criteria includes proximal humeral fractures with one-part or two-part; pathologic fractures or refractures; pseudarthrosis; open fractures; ipsilateral fracture of elbow or distal radius; existing disorders such as paraplegia, disseminated sclerosis, and other related neurological diseases; and acute fracture treated surgically over 21 days after the injury.

### 2.2 Study design

This single-center, prospective, randomized controlled test will be conducted in Tengzhou Central People's Hospital. Consecutive patients with proximal humeral fractures will be stochastic to be dealt with a locking plate or an bone nail. The protocol was admitted by institutional review committee of Tengzhou Central People's Hospital, with the number SD-T-C-9403. The informed consent will be acquired in each patient. This current trial proocol was recorded in the Research Registry, with the number researchregistry6047.

### 2.3 Randomization and blind

**Figure 1:**
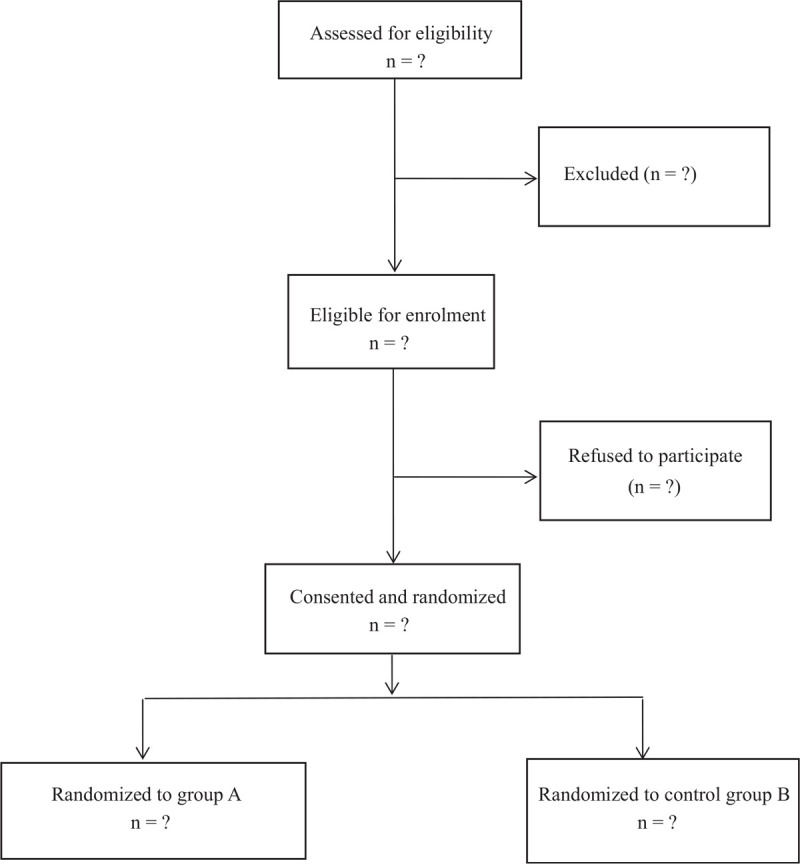
Flow diagram of the study.

After baseline estimation and data capture, patients were stochastically divided into 2 groups by using a computer-created stochastic number table: intramedullary nail and locking plate treatment. Before the surgical operation, the circulating nurse will review the stochastic number table. Patients with even numbers were arranged to the intramedullary nail group and the patients with odd numbers were arranged to the locking plate group. The closed envelope contains the process of treatment assignments. During the whole study, all the researchers could not see the specific grouping of treatment assignments (Fig. 1).

### 2.4 Surgical procedure

The patients will be operated under general anaesthesia. All surgical procedures will be done by the senior surgeon. For both methods, the patient is set in a beach chair position on a light transmitting table with standard armrests.

The traditional deltopectoral approach will be used in patients of locking plate group. Correct repair of the fracture site is performed under C-arm fluoroscopy, and then temporary Kirschner wire or reduction forceps is used to fix the fracture fragments. Locking plate is placed along the lateral edge of biceps groove at the proximal humerus. To prevent subacromial impact, the plate is placed 5 to 8 mm below the tip of the greater tubercle and 2 to 4 mm outside the biceps groove. The correct position of the plates and screws is identified by anteroposterior and axillary fluoroscopy.

The anterolateral approach will be used in patients of intramedullary nail group. A 4-cm-long incision will be made lateral to the acromion parallel to the Langer line. The insertion point was situated at the top of the humeral head, medial to the major tubercle, and lateral to the internodular sulcus. A Proximal Humeral Nail will be inserted without reaming after the fracture is fully reduced. The distal and proximal screws are implanted with assistance. Then the rotator cuff is restored, and the incision is sutured the absence of vacuum drainage.

### 2.5 Rehabilitation protocol

Two groups will use the same postoperative rehabilitation protocol. The outpatient physiotherapy program is arranged at 3 intervals, 10 times each time, lasting for 25 minutes a day. With the help of physiotherapists, passive protected area exercises begin on the first day after surgery. The limb was protected with sling for 4 weeks. Four to six weeks after the operation, they begin comprehensive active and active auxiliary exercise training. Intensive training began 3 months after operation. The patients are followed up at 2 weeks, 6 weeks, 12 weeks and every three months thereafter. The fracture healing and the cervical trunk angle are evaluated by radiology.

### 2.6 Postoperative outcome evaluation

Clinical outcomes include Intraoperative blood loss, operation time, Constant-Murley score (CMS), Disability, Arm, Shoulder and Hand (DASH) score, shoulder range of motion (such as external rotation), and postoperative complications. CMS is a multiple item functional scale used to assess pain, daily activities, range of motion, and shoulder strength. The range of the score is 0–100, indicating the worst and best shoulder function. DASH score is sensitive, reliable and effective in fracture patients, which is broadly applied to evaluate the postoperative outcomes of these patients. It consists of 30 questions, converted into one of 100 points. A higher score indicates greater disability. Image results will be obtained on two planes, and postoperative implant location, secondary humeral head displacement, osteonecrosis, nodule absorption, and secondary fixation failure will be evaluated. Implant malposition refers to the suboptimal implant position in postoperative imaging, without mechanical influences for shoulder function. Secondary fixation failure refers to severe loss of reduction during follow-up.

### 2.7 Statistical analysis and sample size calculation

Continuous data are expressed as mean and standard deviation, and classified data are expressed as absolute numbers and percentages. Student *t* test was used for continuous variables in normal distribution, and Mann Whitney *U* test was used for non-normal distribution. Categoric variables were dealt applying the χ^2^ test or Fisher exact test. The total number of complications was dealt considering the number of possible circumstances. The significance level was defaulted as *P* < .05.

The sample size was calculated based on an average difference of 10% between groups with constant Murley scores adjusted for age and gender. The standard deviation was 10 points, α was 5%, and the power was 80%. The sample size of 25 patients was required for each study group. To compensate for potential withdrawal during the follow-up period, each group included at least 32 patients.

## 3 Discussion

More than 50% of patients over the age of 60 developed complex three-part and four-part of proximal humerus fractures. Because of rotator cuff displacement, humeral head devascularization and osteoporosis, the management of these fractures is very difficult. Therefore, we designed a prospective randomized controlled trial of intramedullary nailing and locking plate in the treatment of three-part and four-part proximal humeral neck fractures in patients aged 55 to 80 years. Our aim was to compare functional outcomes, imaging findings, and complications between the 2 groups.

## Author contributions

**Conceptualization:** Hua Song

**Data curation:** Hua Song, Mingming Wang

**Formal analysis:** Hua Song, Mingming Wang

**Funding acquisition:** Weidong Mu

**Investigation:** Hongyang Du

**Project administration:** Hongyang Du

**Resources:** Weidong Mu

**Software:** Mingming Wang

**Supervision:** Weidong Mu

**Validation:** Mingming Wang

**Visualization:** Hongyang Du

**Writing – original draft:** Hua Song

**Writing – review & editing:** Weidong Mu
